# Infectious agents and progression from Barrett’s oesophagus to oesophageal adenocarcinoma: a nested case-control study

**DOI:** 10.1038/s41416-025-03003-7

**Published:** 2025-04-08

**Authors:** Talita H. A. de Oliveira, Lesley A. Anderson, Stephanie G. Craig, Helen G. Coleman, Tarik Gheit, Sandrine McKay-Chopin, Jacqueline Jamison, Damian T. McManus, Christopher R. Cardwell, Victoria Bingham, Brian T. Johnston, Jacqueline A. James, Andrew T. Kunzmann

**Affiliations:** 1 Patrick G. Johnston Centre for Cancer Research, Queen’s University Belfast, Belfast, Antrim United Kingdom; 2 Aberdeen Centre for Health Data Science, University of Aberdeen, Aberdeen, United Kingdom; 3 Centre for Public Health, Queen’s University Belfast, Belfast, Antrim United Kingdom; 4 International Agency for Research on Cancer-World Health Organization, Lyon, France; 5 Northern Health and Social Care Trust, Antrim, Antrim United Kingdom; 6 Institute of Pathology, Queen’s University Belfast, Belfast, Antrim United Kingdom; 7 Precision Medicine Centre, Queen’s University Belfast, Belfast, Antrim United Kingdom; 8 Belfast Health and Social Care Trust, Belfast, Antrim United Kingdom; 9 Northern Ireland Biobank, Queen’s University Belfast, Belfast, Antrim United Kingdom

**Keywords:** Risk factors, Tumour virus infections

## Abstract

**Background:**

A causal role of high-risk HPV in oesophageal adenocarcinoma development has been hypothesised, but longitudinal evidence is limited. This study aims to investigate a potential causal role of infectious agents in the malignant progression of Barrett’s oesophagus.

**Methods:**

Using a retrospective nested case-control study design, index Barrett’s biopsies were retrieved for individuals within the Northern Ireland Barrett’s oesophagus register who subsequently progressed to oesophageal adenocarcinoma (*n* = 150) and matched non-progressors (*n* = 298). Index Barrett’s biopsies were assessed for the presence of 142 infectious agents by multiplex polymerase chain reaction using the Luminex platform. RNA in-situ hybridisation assessed persistent transcriptional activity in subsequent tissue samples, for infectious agents detected more frequently in progressors.

**Results:**

High-risk HPV genotypes (HPV16 and HPV18) were only identified in the index biopsies of progressors but not non-progressors (4% [5/150] versus 0% [0/298], *P* = 0.004), though no signs of persistence or transcriptional activity were observed in subsequent tissue. Prevalence of infections did not differ between progressors and non-progressors for any other infectious agents, including Helicobacter Pylori and Herpes.

**Conclusion:**

Despite a higher prevalence of high-risk HPV in progressors than non-progressors, no evidence of transcriptionally active high-risk HPV was observed in subsequent samples, indicating presence in Barrett’s is likely non-causal.

## Introduction

Infectious agents are estimated to cause up to 20% of cancers worldwide [1]. HPV subtypes, in particular types 16 and 18, are considered of high oncogenic risk (high-risk HPVs) and were identified as group 1 carcinogens, with an established role in cervical and oropharyngeal cancers [2–4]. The role of infectious agents in oesophageal cancers is less clear, though a potentially causal role has been hypothesised, for both oesophageal squamous cell carcinoma [5] and oesophageal adenocarcinoma [6, 7]. Demonstrating a temporal association, showing the presence of an oncogenic type of HPV and active expression of viral oncogene transcripts are important aspects in establishing a causal relationship [8].

Barrett’s oesophagus is an established premalignant lesion to oesophageal adenocarcinoma, where squamous epithelium is replaced by columnar epithelium [9]. On average, there is a low annual risk of progression to oesophageal adenocarcinoma (~0.5%), which means that regular surveillance endoscopies may not be cost-effective for all individuals. Identifying causes of progression of Barrett’s oesophagus, could be used to inform surveillance, treatment and preventive strategies [10, 11].

Most research surrounding the relationship between viral infectious agents and oesophageal adenocarcinoma, or Barrett’s oesophagus, has centred on HPV infections, and lately, scarce evidence appeared on H. pylori infection and oesophageal cancer development [4, 12]. Our systematic review indicated that HPV was detectable in Barrett’s oesophagus and oesophageal adenocarcinoma tissue samples, though the estimates of prevalence varied considerably [13]. Concerns were raised about the quality of some primary studies, as many studies were small, had unknown sensitivity/specificity for HPV detection and were deemed to be at risk of cross-contamination, which may have exaggerated detection [13]. The evidence relating to other infectious agents was more limited [13].

A causal role of high-risk HPV (or other infectious agents) has not been convincingly assessed. Our review indicated a higher prevalence of HPV DNA in oesophageal adenocarcinoma cases than controls, though the cross-sectional study designs hinder causal interpretations [6, 7]. Longitudinal studies using strict anti-contamination procedures to assess the presence of infectious agents prior to, and following, tumour formation, may better investigate a potential causal role in oesophageal adenocarcinoma development.

The aim of the current study was (1) to assess the prevalence of various infectious agents, including HPV, in Barrett’s oesophagus progressors and matched non-progressors using a strict anti-cross-contamination procedure; (2) to assess whether there is an association with subsequent progression to oesophageal adenocarcinoma; and (3) assess transcriptional activity in subsequent tumour/Barrett’s tissue to investigate persistence indicative of a potential causal role.

## Methods

### Study population

Participants were identified from the Northern Ireland Barrett’s oesophagus Register (NIBR). The NIBR is a unique population-based cohort of over 12,000 individuals with Barrett’s oesophagus aged 16 years or over from throughout Northern Ireland (population 1.9 million) diagnosed between 1993 and 2013 (for details on NIBR establishment and criteria see Bhat S. et al. 2011) [14, 15]. The date of the first biopsy showing Barrett’s oesophagus was taken as the date of diagnosis. Individuals with an index Barrett’s biopsy without dysplasia or previous oesophageal cancer were selected and included if the sample was available in the Northern Ireland Biobank.

### Identification of cases and matched controls

Progressors were identified via linkage of the NIBR to the population-based Northern Ireland Cancer Registry (NICR) using patient forename, surname, date of birth, and where available, the unique Health and Social Care (HSC) number. Progressors were classed as individuals within the NIBR who had progressed to adenocarcinoma or histologically unspecified tumours of the oesophagus (International Classification of Diseases V.10 (ICD10 C15.5 or C15.9)) until end 2013. Individuals diagnosed with oesophageal adenocarcinoma within 6 months of Barrett’s oesophagus diagnosis were excluded as they were considered to be prevalent cancers.

Progressors were individually matched to up to three non-progressors, who had Barrett’s oesophagus (within NIBR) but had not progressed to oesophageal adenocarcinoma by end 2013 and had the same length of follow-up (up to 31st December 2013 or date of death). Matching criteria were based on age at Barrett’s oesophagus diagnosis (<50, 50–59, 60–69, 70–79, 80+ years), sex, year of incident Barrett’s oesophagus diagnosis (selecting within the same year initially before expanding to within five years sequentially), the presence of specialised intestinal metaplasia and HSC Trust (Belfast, Northern, Southern and Western). The two closest matched non-progressors based on year of Barrett’s oesophagus diagnosis were selected first, with the third non-progressor used if samples for either of the other two controls were unavailable.

### Specimen collection

Archived Formalin Fixed Paraffin Embedded (FFPE) tissue blocks were retrospectively obtained from the HSC Trusts (15/NI/0233), or the Northern Ireland Biobank (15/NI/0233; NIB15-0146) (which covered Belfast and South Eastern HSC Trusts at the time of the study) [16]. The blocks (index biopsy or next available interim sample) were sent to the Cellular Pathology laboratories, Antrim area Hospital, Northern HSC Trust for sectioning.

Sections (one 1 µ, four 5 µ and six 10 µ) were cut using a microtome to obtain thin rolls of tissue from the FFPE Barrett’s oesophagus tissue under a strict protocol to decrease risk of cross-contamination whereby the top surface of the FFPE block was removed, the microtome and all other apparatus were cleaned with hypochlorous acid disinfectant solution and isopropanol before use and after each block was cut using a new blade each time. To assess the potential for cross-contamination, known HPV positive and negative cervical tissue samples were sent to the Northern Health & Social Care trust laboratories for sectioning, before being returned to International Association for Research in Cancer (IARC) Lyon, France for HPV testing, to identify and rectify any cross-contamination risk.

Two of the 5 μ Sections were then histologically inspected by an expert pathologist to confirm the presence of columnar epithelium, the lack of prevalent adenocarcinoma and ensure specialised intestinal metaplasia status classification was correct.

### Identification of infectious agents from biopsy specimens

Three 10 µ sections were sent in new, disinfected tubes to the International Association for Research in Cancer (IARC), Lyon, France. Human DNA was extracted from the FFPE samples using the QIAamp DNA FFPE Tissue Kit (PN 564646, QIAGEN UK Lts., Surrey, UK) following the manufacture’s protocol. The extracted DNA was quantified using the NanoDrop 2000 Spectrophotometer (Fisher Scientific UK Ltd, Loughborough, UK), and the integrity of the DNA assured by the amplification of Beta -globin gene. Only samples positive for the beta-globin gene were included in the primary analysis, however secondary analyses including samples with undetectable beta-globin was also performed to assess sensitivity of the results to missing data and added in the supplementary material.

The infectious agents DNA was amplified by multiplex polymerase chain reactions (PCR) protocol, (PMID: 16757593),and separate typing assays using bead-based hybridization (LUMINEX® technology) were run, each with a beta-globin control, for mucosal/alpha HPVs (*n* = 21); Beta HPVs (*n* = 46); Gamma HPVs (*n* = 52); Polyoma (*n* = 13) and Herpes (*n* = 10) viruses; Chlamydia Trachomatis and H. pylori (PMID: 25478862, PMID: 16455905, PMID: 19864475, PMID: 28480229, PMID: 27227411, PMID: 31406502) (Table 1). For H. pylori, both amplification of 16S rRNA and ureA gene were used to detect the bacteria. To ensure systematic contamination was not present, positive and negative controls were included at each step of the PCR process.

**Table 1:** List of 142 human infectious agents included in a Luminex-based platform.

Infectious Agent	Subtypes
Mucosal high-risk HPV types^a^ (*n* = 19)	16, 18, 26, 31, 33, 35, 39, 45, 51, 52, 53, 56, 58, 59, 66, 68, 70, 73 and 82
Mucosal low-risk HPV types (*n* = 2)	6 and 11
Cutaneous gamma HPV (*n* = 52)	4, 48, 50, 60, 65, 88, 95, 101, 103, 108, 109, 112, 116, 119, 121, 123, 126, 127, 128, 129, 130, 131, 132, 133, 134, 148, 149, 156, 161, 162, 163, 164, 165, 166, 167, 168, 169, 170, 171, 172, 173, 175, 178, 179, 180, 184, 197, 199, 200, 201, 202, SD2
Cutaneous beta HPV types (*n* = 46)	5, 8, 9, 12, 14, 15, 17, 19, 20, 21, 22, 23, 24, 25, 36, 37, 38, 47, 49, 75, 76, 80, 92, 93, 96, 98, 99, 100, 104, 105, 107, 110, 111, 113, 115, 118, 120, 122, 124, 143, 145, 150, 151, 152, 159, 174
Polyomaviruses (*n* = 13)	BKPyV, WUPyV, KIPyV, MCPyV, JCPyV, HPyV6, HpyV7, HPyV9, HPyV10, TSPyV, HPyV12, LIPyV and SV40,
Herpes Viruses (*n* = 10)	HSV1, HSV2, EBV1, EBV2, CMV, HHV6A, HHV6B, HHV7, HHV8 (KSHV), HZV
Other infectious agents (*n* = 3)	Helicobacter Pylori, Chlamydia trachomatis, Schistosoma spp.

^a^High-risk subtypes were determined at time of protocol and formal definitions may be subject to change. *HPV* Human papilloma virus, *BKV* BK polyomavirus, *WUV* Washington University virus, *KIV* Karolinska Institute virus, *MCV* Merkel cell polyomavirus, *JCV* John Cunningham virus, *HPyV* Human polyomavirus, *TSV* Taura syndrome virus, *SV40* Simian virus 40, *HSV* herpes simplex virus, *EBV* Epstein-Barr virus, *CMV* Cytomegalovirus, *HHV* Human Herpesvirus, *KSHV* Kaposi’s sarcoma-associated herpesvirus, *HZV* Herpes zoster virus.

### RNA in-situ hybridisation (ISH)

If type-specific viral DNA was more prevalent in progressors and non-progressors (*p* < 0.05), subsequent tumour (or last available Barrett’s) biopsy samples were sought within the NI Biobank, using NICR records. The NI Biobank team used a strict anti-cross contamination procedure for sectioning.

HPV RNA-ISH was conducted for samples for which suitable mRNA integrity had been detected using positive (PPIB) and negative (DAPB) reagent controls (Advanced Cell Diagnostics, Newark, CA, USA; catalog numbers: 313908 for PPIB and 312038 for DapB). HPV RNA-ISH was undertaken using RNAscope 2.5 LSx automated assay for use of the Leica Bond RX with genotype specific HPV E6/E7 mRNA detection kit (Advanced Cell Diagnostics; catalog numbers: 322700 for RNAscope™ 2.5 LSx Reagent Kit-BROWN, 311528 for HPV16 and 311538 for HPV18) within the Precision Medicine Centre at Queen’s University Belfast, as previously described [17]. Consultant pathologists identified suitable tumour tissue locations and assessed histological characteristics consistent with HPV activity in other tissue types. Samples positive for PPIB and negative for DAPB, were considered HPV RNA-ISH positive if the tissue demonstrated punctate dots of brown reaction product specifically localizing within the malignant/dysplastic cells.

### Sample size

Prior to the study, a simplified sample size calculation indicated that a study with 150 progressors and 300 non-progressors would have 80% power to detect an Odds Ratio (OR) of 2.00 between an infectious agent and risk of oesophageal adenocarcinoma progression, assuming 18% positivity in Barrett’s oesophagus patients (based upon background rates in the healthy Northern Ireland population). The power would be greater for more common infections but reduced for less common infections.

### Statistical analysis

Comparisons in baseline characteristics were made between progressors and non-progressors using chi-square tests for categorical variables and *t*-tests for continuous variables. Conditional logistic regression, without additional adjustments, compared prevalence of common infectious agent DNA between progressors and matched non-progressors. As conditional logistic regression was not possible for all analyses due to zero values and excluded Beta-globin positive samples due to matching rules, Fisher’s exact tests were calculated. Analyses were conducted using Stata/IC statistical software (version 15.1, College Station, TX, USA).

## Results

### Participant characteristics

From 12,457 individuals registered in the NI Barrett’s Register from 1993 to 2013, 197 progressors to oesophageal adenocarcinoma were identified, and 180 progressors were matched to 358 non-progressors. Of these, samples from 150 progressors and matched 298 non-progressors were available for viral DNA analysis.

Participant characteristics are shown in Table 2. Age, sex, HSC trust, specialised intestinal metaplasia status and year of diagnosis were similar between progressors and matched non-progressors, as expected. Median age for progressors was 62.7 years (Interquartile range [IQR]: 54.2–71.4 years). Most progressors (70%) were male, since it is one of the risk factors of Barrett’s oesophagus development and had specialised intestinal metaplasia (78.5%) (Table 2). Median time from Barrett’s index biopsy to progression was 6.0 years (IQR: 3.0–8.6 years)

**Table 2:** Demographic information of Barrett’s oesophagus patients selected for this study.

	Non-progressors *N* (%)	Progressors *N* (%)
Total	298	150
Age at Barrett’s diagnosis (years)		
<50	40 (13.4)	20 (13.3)
50– < 60	74 (24.8)	37 (24.7)
60– < 70	100 (33.6)	49 (32.7)
70– < 80	67 (22.5)	35 (23.3)
80+	17 (5.7)	9 (6.0)
Sex		
Women	85 (28.5)	44 (29.3)
Men	213 (71.5)	106 (70.7)
Healthcare Trust		
Belfast	181 (60.7)	91 (60.7)
North	48 (16.1)	29 (19.3)
South	26 (8.7)	11 (7.3)
West	43 (14.4)	19 (12.7)
Specialised intestinal metaplasia		
No/unknown	62 (20.8)	32 (21.3)
Yes	236 (79.2)	118 (78.7)
Barrett’s diagnosis year		
1993–1995	67 (22.5)	28 (18.7)
1996–2000	104 (34.9)	52 (34.7)
2001–2005	102 (34.2)	52 (34.7)
2006–2010	25 (8.4)	18 (12.0)

### Beta-globin detection

Beta-globin was detected, indicating adequate DNA integrity, in a similar proportion of progressors and non-progressors, though positivity declined over subsequent assays (mucosal HPV, followed by beta HPV; gamma HPV; and polyoma and herpes virus assays, respectively) (Table 3 and Supplementary table 1).

**Table 3:** Prevalence of infectious agents detected in Barrett’s oesophagus patients.

	Progressors	Non-progressors	*P*-value
Any HPV			
No	117	244	
Yes	8 (6.4%)	14 (5.4%)	0.82
High-risk HPV			
No	118	256	
Yes	5 (4.1%)	0 (0%)	0.004
Gamma HPV			
No	48	102	
Yes	0 (0%)	7 (6.4%)	0.15
Herpes virus (any)			
No	48	100	
Yes	9 (15.8%)	18 (15.3%)	0.96
*H. pylori*			
No	51	108	
Yes	12 (19.1%)	30 (21.7%)	0.71

### Prevalence of total and low-risk HPV

The prevalence of HPV overall was similar between progressors and non-progressors (HPV prevalence=6.4% in progressors and 5.4% in non-progressors, Supplementary table 1), including in conditional logistic regression analyses (Odds ratio:1.07, 95% CI: 0.49–2.30, Table 4)

**Table 4:** Conditional logistic regression analyses of the odds of positivity for HPV, Herpes viruses and *H. pylori* in Barrett’s oesophagus patients using the Luminex assay.

	Progressors	Non-progressors	Odds ratio (95% confidence limits)	*P*-value
Any HPV				
No	114	208		
Yes	8 (6.6)	11 (5.0%)	1.35 (0.52–3.48)	0.54
Herpes virus (any)				
No	37	52		
Yes	5 (11.9%)	8 (13.3%)	0.94 (0.29–3.05)	0.92
H. pylori				
No	43	63		
Yes	6 (12.2%)	10 (13.7%)	0.85 (0.27–2.65)	0.77

Only individuals with detectable beta-globin were eligible. Sets without an eligible progressor and/or non-progressor were dropped. Conditional logistic regression was performed without additional adjustments.

Gamma HPV was only observed in non-progressors (*p* = 0.15, Table 3). Beta HPV, low-risk mucosal HPV types, - and combined mucosal HPV types were uncommon, with similarly low prevalence between progressors and non-progressors (data not shown due to low numbers).

### Prevalence of High-risk HPV DNA and RNA

High-risk HPV types 16 and 18 were detected in five samples, all of which were progressors (4.1% progressors vs 0% of non-progressors), which reflected a statistically significant difference (Fisher’s *p* = 0.004, Table 3). HPV RNA ISH positivity was not detected in subsequent tumor/Barrett’s tissue samples from any of the five individuals with high-risk HPV DNA detected in their index Barrett’s tissue (supplementary fig. 1). Sample integrity, via PPIB positivity and DAPB negativity was supported for all five samples. No distinct/unusual histological features in the subsequent tissue were observed during histological review by an expert pathologist.

### Prevalence of Herpes and polyomavirus

Herpes virus DNA had a similar detection rate in progressors (*n* = 9, 15.8%) and non-progressors (*n* = 18, 15.3%) (Tables 3 and 4). EBV (type 1 or 2) was also detectable in a similar proportion of progressors and non-progressors (data not shown due to small numbers).

Positivity for polyomaviruses was uncommon in both progressors and non-progressors (< 5 in each).

### H. pylori

H. pylori (overall, if either the 16S rRNA or ureA gene was amplified) was detected in 42 Barrett’s samples, with a similar detection rate in progressors (*n* = 12, 19.1%) and non-progressors (*n* = 30, 21.7%) (Table 3).

In conditional logistic regression analyses, the odds of positivity for H. pylori (16S and/or urea) were similar between progressors and non-progressors (Table 4 and Supplementary Table 1).

### Other infectious agents

No other evaluated agents (Chlamydia T. and Schistosoma spp.) were detected in the samples.

## Discussion

This nested case-control study within a population-based disease registry, using a strict anti-contamination procedure found a low prevalence of HPV DNA in index Barrett’s biopsy tissue. Prevalence of HPV, Herpes viruses or H. pylori appeared to be similar between progressors and non-progressors, though herpes virus and H. pylori were more commonly identified than HPV. High-risk HPV DNA types 16 and 18) was only identified in Barrett’s oesophagus tissue from progressors (~4%), though no signs of transcriptional activity were observed in subsequent tissue samples.

In the current study, we identified a lower, but non-zero, prevalence of HPV DNA in Barrett’s oesophagus tissue (~5–7%) compared to previous pooled estimates (~25%) [13]. Our previous pooled estimates indicated considerable between-study heterogeneity in HPV prevalence estimates for Barrett’s oesophagus (ranging from 0% to 96.4%), and oesophageal adenocarcinoma [13], which has been noted for other cancer types [18, 19]. The discrepancy in prevalence could be due to ethnical and cultural differences (e.g. sexual behaviour) between populations, however, a population-based study in Northern Ireland showed 68% of HPV-positive cervical tissue [20], suggesting that even with a high rate of positivity in the studied population, HPV is not infecting the oesophageal lower third in the same proportion. Avoiding cross-contamination is also a key issue for accurately assessing the prevalence of HPV. Cross-contamination could exaggerate prevalence in some prior studies, judged to be at a high risk of cross-contamination in a previous systematic review [13]. The current study used a strict anti-cross contamination procedure; a high specificity technique for confirming the presence of HPV; and used samples from a population-based registry [21], which may all facilitate more accurate estimation. However, we cannot rule out cross-contamination entirely. Similarly, differences in prevalence of HPV have been noted for other cancers and may also explain some of the differences in HPV DNA prevalence observed in the current study.

A potential causal role of high-risk HPV has been proposed previously, though most previous studies have used a cross-sectional design unsuitable for establishing a potential causal role [6, 22–24]. Rajendra et al., found transcriptional activity of HPV oncogenes E6 and E7 in oesophageal adenocarcinoma tissue, and separate Barrett’s tissue, which is consistent with a carcinogenic role, but couldn’t assess whether the activity preceded tumour formation [6, 22].

The current study’s nested case-control design indicated that high-risk HPV DNA may be present prior to tumour formation and was associated with risk of progression. However, assessment of subsequent tumour tissues from individuals with high-risk HPV DNA in their index Barrett’s tissue, using a high sensitivity, high specificity RNA assay, did not support a persistent, active role of HPV. Similarly, histological review did not reveal any obvious indicators of HPV-related disease, though the limited sample size may preclude firm conclusions. Therefore, we did not find strong evidence to support a causal role of high-risk HPV in the progression from Barrett’s to oesophageal adenocarcinoma.

The high risk of progression in high-risk HPV positive Barrett’s, observed in our study could support investigations of prognostic utility of HPV testing. However, the low sample size, rareness of high-risk HPV positive and unclear rationale for the discrepancy, may hinder efforts. Confounding, perhaps by immune dysfunction in higher risk lesions, is possible, but is not supported by overall similarity of other viral types, and the unexpected [25, 26] slightly lower prevalence of gamma HPV in progressors. One potential explanation is an inadvertent batch effect of progressor and non-progressor tissue, based on the order of receiving tissue samples (despite blinding of investigators to progression status).

The lack of a difference in prevalence of DNA from herpes viruses, including EBV, do not support a causal role, which appears to be consistent with the limited available evidence [13]. Collectively, herpes viruses were present in ~15% of Barrett’s samples and a role in Barrett’s formation, rather than progression cannot be entirely ruled out since Herpes viruses can cause oesophagitis and was related to oesophageal squamous cell carcinoma development [27].

H. pylori has been postulated as a potentially protective factor against gastroesophageal reflux disorder [28–30], which may reduce risk of oesophageal adenocarcinoma. A previous meta-analysis of observational studies indicated that H. pylori infections were associated with a reduced risk of oesophageal adenocarcinoma [30]. However, a recent study did not find an increased risk of oesophageal adenocarcinoma after H. pylori eradication therapy [31]. Our findings are more consistent with the latter study, as overall there was only a modest, not statistically significant reduction in H. pylori positivity in progressors compared to non-progressors. The discrepancy may reflect a stronger role of gastroesophageal reflux in Barrett’s formation than oesophageal adenocarcinoma [32]. H. pylori’s role in Barrett’s and oesophageal adenocarcinoma remains inconclusive though, and more studies to elucidate the mechanisms by which H. pylori may have a protective effect on the oesophagus are required [33].

### Strengths & limitations

One of the major strengths of the current study was the use of a high-quality detection method for the presence of viral agents with a strict protocol to avoid cross-contamination. The nested-case control study design gives stronger evidence than previous studies that the infection precedes malignancy, but reverse causality, or transient infections cannot be fully ruled out. The sensitivity of the multiplex assays was also high, with prior studies demonstrating detection sensitivity to 10 copies of the viral genome, with strong reproducibility between runs. The high sensitivity was particularly important given the age of the samples. Whilst matching can reduce confounding, a limitation was a lack of information on other risk factors (such as smoking status) which could be a source of residual confounding. However, as some sources of confounding may overlap between HPV types, the lack of association between low-risk HPV types and progression to oesophageal adenocarcinoma, may reduce concerns over considerable confounding, or suggest confounding is specific to high-risk HPV types. Another limitation was the age of the samples, some of which were more than 20 years old. However, only samples positive for beta-globin amplification were included in the analysis, which should ensure the denominator for prevalence calculations was within samples with adequate DNA integrity.

From the three suggested criteria to determine HPV causal role in cancer formation [8], only two were analysed in the present study, however since no transcriptional activity was found in tumour samples, there was no need to further assess the expression of p16. Future research focused on infectious agents and molecular aberrations, such as p53, pRb and p16 would be interesting to check if there is any relationship with cancer formation and infection.

## Conclusions

The current study found that the prevalence of HPV in Barrett’s oesophagus tissue from index biopsies was lower than previous estimates identified in our systematic review [13]. High-risk HPV was only infrequently identified in index Barrett’s tissue in individuals who subsequently progressed to oesophageal adenocarcinoma. However, there were no signs of transcriptional activity in subsequent tissue samples to support a carcinogenic role and infections may have been transient or evaded anti-contamination efforts. The similarity in prevalence of other infectious agents does not support an infectious component of progression from Barrett’s to oesophageal adenocarcinoma, though other studies to investigate a role in Barrett’s formation may be required for H. pylori and Herpes virus.

## Disclaimer

Where authors are identified as personnel of the International Agency for Research on Cancer/World Health Organization, the authors alone are responsible for the views expressed in this article, and they do not necessarily represent the decisions, policy, or views of the International Agency for Research on Cancer/World Health Organization.

## Supplementary information

Supplementary material

## Data Availability

The data held within the Northern Ireland Cancer Registry and NIBR is subject to strict confidentiality rules (https://www.qub.ac.uk/research-centres/nicr/CancerInformation/requests/), which does not allow direct data sharing due to the sensitive nature of the data. Researchers wishing to use this resource can contact the Northern Ireland Cancer Registry, whose information is available at: http://www.qub.ac.uk/research-centres/nicr/. The quantitative data generated in this study is available via direct contact of the corresponding author.
