# Supplementary material for: Infectious agents and progression from Barrett’s oesophagus to oesophageal adenocarcinoma: a nested case-control study
Source: Br J Cancer. 2025 Apr 8;132(11):1050–5. doi: 10.1038/s41416-025-03003-7 (PMC12119926; doi:10.1038/s41416-025-03003-7)
Supplement: Supplementary file 1 — Supplementary material [file 41416_2025_3003_MOESM1_ESM.docx]

**Supplementary material**

Supplementary Table 1. Conditional logistic regression analyses comparing positivity of infectious agent DNA between progressors and matched non-progressors, regardless of Beta-globin positivity.

|  | Progressors | Non-progressors | P-Value^1^ | Odds ratio  (95% confidence limits) | P-value^2^ |
| --- | --- | --- | --- | --- | --- |
| Any HPV |  |  |  |  |  |
| No | 139 | 277 |  |  |  |
| Yes | 11 (7.3%) | 21 (7.1%) | 1.00 | 1.07 (0.49-2.30) | 0.87 |
|  |  |  |  |  |  |
| High-risk HPV |  |  |  |  |  |
| No | 145 | 297 |  |  |  |
| Yes | 5 (3.3%) | 0 (0%) | 0.004 | - | - |
|  |  |  |  |  |  |
| Herpes virus (any)^2^ |  |  |  |  |  |
| No | 134 | 267 |  |  |  |
| Yes | 16 (10.7%) | 29 (9.7%) | 0.87 | 1.10 (0.58-2.10) | 0.76 |
|  |  |  |  |  |  |
| H. Pylori^3^ |  |  |  |  |  |
| No | 128 | 241 |  |  |  |
| Yes | 22 (14.7%) | 57 (19.1%) | 0.29 | 0.69 (0.39-1.21) | 0.19 |

^1^ Fischer’s Exact test.

^2^ Samples available for 150 progressors and 296 non-progressors for Herpes virus analysis

^3^ Either *H. Pylori* 16S or *H. pylori* Urea positive


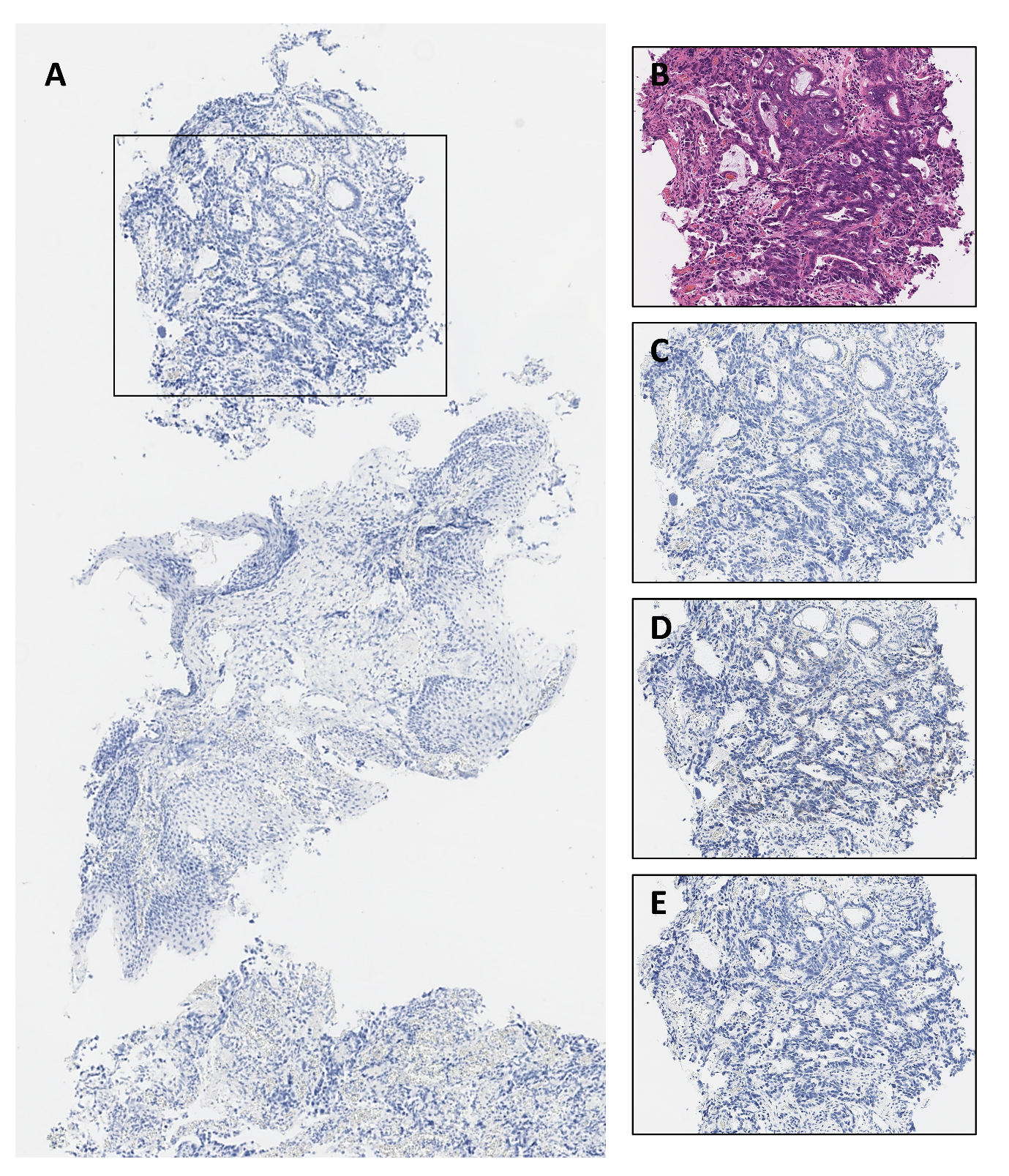


Supplementary Figure 1. Representative Brightfield image of oesophageal adenocarcinoma demonstrating the lack of persistent HPV infection using RNA ISH. No positivity for genotype-specific HPV infections was detected in subsequent tumour tissue samples from the five patients with high-risk HPV DNA detected in their index Barrett’s tissue. A) HPV RNA ISH at 4X; B) H&E at 20x; C) HPV RNA ISH at 20x showing a negative result; D) PPIB positive control, at 20x demonstrating acceptable tissue/RNA quality for HPV RNA ISH analysis; and, E) DAPB negative control, at 20x demonstrating acceptable tissue/RNA quality for HPV RNA ISH analysis.
